# Massive Atomic Diversity: a compact universal dataset for atomistic machine learning

**DOI:** 10.1038/s41597-025-06109-y

**Published:** 2025-11-21

**Authors:** Arslan Mazitov, Sofiia Chorna, Guillaume Fraux, Marnik Bercx, Giovanni Pizzi, Sandip De, Michele Ceriotti

**Affiliations:** 1 Laboratory of Computational Science and Modeling, Institut des Matériaux, École Polytechnique Fédérale de Lausanne, Lausanne, 1015 Switzerland; 2 PSI Center for Scientific Computing, Theory, and Data, and National Centre for Computational Design and Discovery of Novel Materials (MARVEL), PSI, Villigen, 5232 Switzerland; 3 BASF SE, Carl-Bosch-Straße 38, Ludwigshafen, 67056 Germany

**Keywords:** Atomistic models, Electronic structure

## Abstract

The development of machine-learning models for atomic-scale simulations has greatly benefited from the large databases of materials and molecular properties, computed using electronic-structure calculations. Recently, these databases enabled the training of “universal” models that aim to make accurate predictions for arbitrary atomic geometries and compositions. However, many of these databases were originally designed for materials discovery, focusing primarily on equilibrium structures. Here, we introduce a dataset designed to train machine-learning models to make reasonable predictions for arbitrary structures. Starting with relatively small sets of stable structures, we built the dataset aiming to achieve “massive atomic diversity” (MAD) by aggressively modifying these structures and utilizing highly consistent electronic-structure settings for property calculations. Despite containing fewer than 100,000 entries, the MAD dataset has already enabled the training of universal interatomic potentials that rival those trained on datasets containing two to three orders of magnitude more data. We detail the design philosophy of the dataset and introduce low-dimensional structural latent space descriptors that can be used as a general-purpose materials cartography tool.

## Background & Summary

The introduction of large-scale, open-access materials databases has significantly accelerated computational materials science and discovery^1^. They offer vast repositories of atomic structures and computed or experimentally measured properties of organic and inorganic compounds, facilitating high-throughput screening for many materials-discovery applications. Among these, the databases of electronic structure calculations serve as a particularly important source of data for atomistic modeling, providing a robust and consistent way of exploring structure-property relations for a wide range of materials, including those that have never been experimentally realized^2–11^. Despite these advancements, existing datasets primarily focus on structures at or near the local minima and saddle points of the potential energy surface (PES), limiting their applicability in atomistic simulations that often require exploration of mid- and high-energy configurations. This is particularly important for interatomic potentials — approximations of the PES — which require accurate descriptions of both low- and high-energy states to ensure robustness across a wide range of thermodynamic conditions. Another source of error stems from the presence of inconsistencies in computational settings between different datasets and between different structures within the dataset. For example, some compositions may be treated with different electronic-structure details to tackle known shortcomings of density-functional theory, which, however, means that different portions of chemical space are associated with different PES. Furthermore, most of the existing datasets are focused on either organic or inorganic materials – which is well motivated by the fact that these classes of materials often require different electronic-structure details and cover different energy scales, but restricts the development of universal interatomic potentials capable of handling hybrid systems of various nature and chemical compositions.

To address these challenges, we introduce the Massive Atomic Diversity (MAD) dataset, designed to encompass a broad spectrum of atomic configurations, including both organic and inorganic systems, while being restricted to a small number of structures – which facilitates property estimation with converged settings, and reduces the cost of training new models based on it. By applying systematic perturbations to stable structures and maintaining consistent computational parameters, we aim to provide a coherent structure-energy mapping suitable for training robust, general-purpose machine-learning interatomic potentials that can be used reliably for complex atomistic simulation workflows. In the following sections, we detail the construction methodology of the MAD dataset and analyze its diversity and consistency.

### The MAD dataset

In contrast to most existing datasets for atomistic machine learning, which usually contain stable — or judiciously distorted — configurations of materials, focusing primarily on either inorganic or organic domains, the MAD dataset is based on a different philosophy. It draws inspiration from the *mindless dataset* proposed by Korth and Grimme to benchmark quantum chemistry methods^12^. First, it aims to extend the limits of universality by incorporating both organic and inorganic materials, thus allowing the creation of models capable of performing atomistic simulations in both domains. Second, it systematically extends the coverage of the configuration space by adding relaxed structures, their rattled counterparts, structures with randomized composition, clusters, molecules and surfaces, enabling complex simulation protocols in a broad range of thermodynamic states, including out of equilibrium conditions (see Table 1 for an overview of the different subsets of structures included in MAD). Third, it uses a consistent level of theory across all ab initio calculations to ensure a coherent structure-energy mapping for the included structures. This, unfortunately, means the MAD dataset neglects the description of a few important physical effects, such as magnetism, electron correlations, and dispersion, which can be important for certain types of materials, yet cannot be applied consistently across the MAD dataset. Section 3 gives more details on the first-principles calculations. Last but not least, while maintaining a reasonable descriptive power, the MAD dataset is designed to be lightweight, consisting of fewer than 100,000 structures, thus significantly reducing the total amount of computational resources required for training and making it accessible to a wider community.

**Table 1:** Description of structural subsets used that constitute the MAD dataset.

Subset name	Description	# structures	# atoms
MC3D	Bulk crystals from the Materials Cloud 3D crystals database^42^	33596	738484
MC3D-rattled	Rattled analogs of the original MC3D crystals, with Gaussian noise added to all atomic positions	30044	599675
MC3D-random	Artificial structures from MC3D with randomized atomic species sampled from the list of 85 elements	2800	25095
MC3D-surface	Surface slabs generated from MC3D by cleaving along random low-index crystallographic planes	5589	205185
MC3D-cluster	Nanoclusters (2-8 atoms) cut from MC3D and MC3D-rattled crystals as random atomic environments	9071	44829
MC2D	Two-dimensional crystals from the Materials Cloud 2D database^43,44^	2676	43225
SHIFTML-molcrys	Curated SHIFTML molecular crystals from the Cambridge Structural Database^45,46^	8578	852044
SHIFTML-molfrags	Neutral molecular fragments from the SHIFTML dataset^47^	3241	72120

**Fig. 1:**
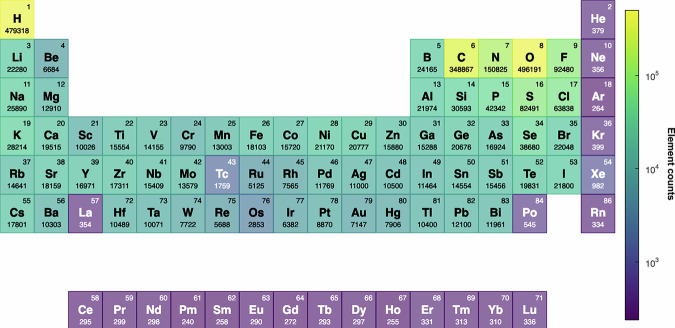
Periodic table indicating the statistical representation of the elements present in the MAD dataset.

As outlined in Ref. ^13^, the MAD dataset contains 95595 structures, containing 85 elements in total (with atomic numbers ranging from 1 to 86, excluding Astatine). The statistical representation of the occurrence of elements across the dataset is presented in Fig. 1. Despite being relatively lightweight, MAD provides a good coverage of the main-block elements, with an over-representation of the first-period elements, that are abundant in the “organic” subsets of MAD. In addition, MAD naturally under-represents noble gases due to their low reactivity and limited occurrence in nature, as well as lanthanides due to technical reasons related to the poor robustness of the underlying DFT calculations – which means that the reference data is likely to be of low quality, and therefore of little practical use except for low-accuracy preliminary studies. More details on this latter issue are provided in Section 3.

**Fig. 2:**
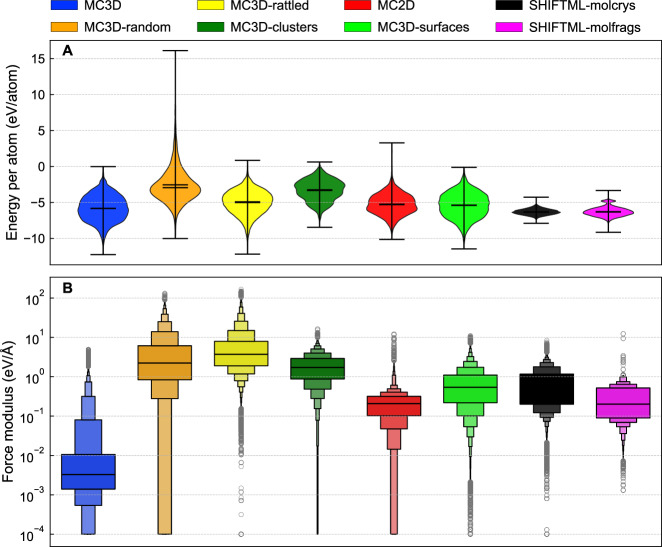
Histograms of energy per atom (top) and force magnitude (bottom) within the different subsets of the MAD dataset.

We further characterize the MAD dataset in terms of the distribution of energy and force values within each of the subsets. As shown in Fig. 2, these values vary quite significantly: the MC3D, MC2D, SHIFTML datasets are based on stable, or low-temperature MD configurations, and have small interatomic forces. In contrast, the MC3D-derived subsets, that are built introducing large distortions in the chemical and structural parameters, cover a much larger energy range, which helps obtain models that are capable of handling highly-distorted, unexpected configurations, and therefore increases the extrapolation capabilities of the trained models.

### A map of the MAD chemical space

To substantiate our claim that MAD covers a broader portion of chemical space than existing datasets, as well as to lay the foundations for a materials dataset characterization framework, we proceed to define a low-dimensional representation of the space covered by MAD. To this end, we need a high-dimensional representation of the structures and a strategy to reduce the feature space to a lower dimensionality (2D or 3D) that can be visualized conveniently.

For the high-dimensional description, we use the last-layer features of the trained PET-MAD model^13^, that provide a 512-sized token that describes each *i* − atom-centered environment in a given structure, ***ξ***(*A*_*i*_). Given that we aim to characterize *structures* rather than environments, we describe each configuration using a 1024-vector obtained by concatenating the entry-wise mean and standard deviation of the environment features

$${\boldsymbol{\Xi }}(A)=[\frac{1}{{N}_{A}}\sum _{i}{\boldsymbol{\xi }}({A}_{i}),\sqrt{\frac{1}{{N}_{A}}\sum _{i}{({\boldsymbol{\xi }}({A}_{i})\,-\,\langle {\boldsymbol{\xi }}({A}_{i})\rangle )}^{2}}].$$

This choice leads to an intensive description (if one replicates a periodic structure, the features remain unchanged), but it is capable of describing the degree of inhomogeneity of a configuration.

**Fig. 3:**
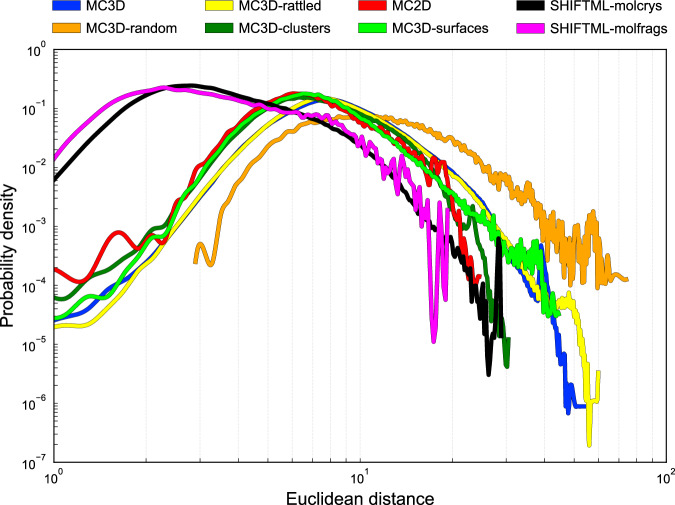
Histogram of the Euclidean distances between structures in the different subsets, computed in the space of high-dimensional PET-MAD features.

A histogram of the Euclidean distances between pairs of configurations within each of the MAD subsets (Fig. 3) demonstrates how the latent features capture the diversity of each subset, with the molecular datasets (that cover a small portion of chemical space) being peaked at small inter-configuration distance, and the more diverse MC3D-derived structures having a tail of large distances – with the highest diversity corresponding to the MC3D-random subset.

**Fig. 4:**
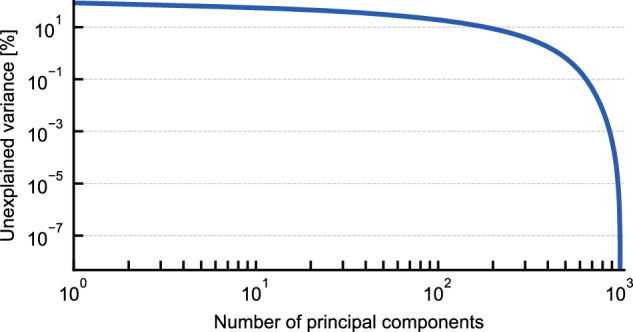
Residual variance as a function of the number of principal components. The steady decrease with more components shows the intrinsically high dimensionality of the dataset.

**Fig. 5:**
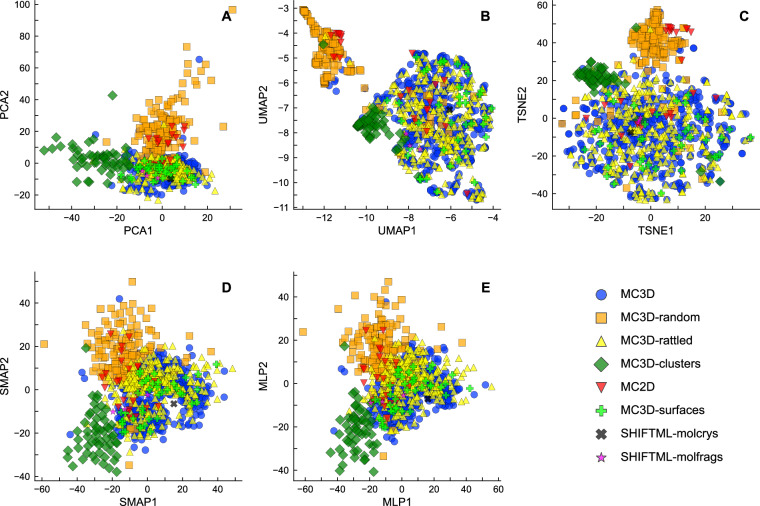
Two-dimensional projections of 1,000 representative MAD structures, selected via farthest-point sampling, based on high-dimensional features from the PET-MAD model. (**A**) PCA, (**B**) UMAP, (**C**) t-SNE, (**D**) Sketch-map projection, and (**E**) MLP-predicted sketch-map projection, learned to map high-dimensional descriptors to 2D space. The axes labels indicate the method used to determine the low-dimensional latent space in each map.

Even though these histograms provide a way to qualitatively measure diversity in the high-dimensional feature space, an intuitive visualization requires performing a dimensionality reduction starting from the feature vectors. A principal component analysis (PCA) shows that the intrinsic dimensionality of the dataset is high, with a slow and smooth decay of the residual variance with the number of components included (Fig. 4). As a consequence, the low-dimensional representation is bound to be lossy and to distort the relation between different structures. For this reason, we compare several non-linear dimensionality-reduction algorithms (Fig. 5). We look for a projection that separates the different parts of PET-MAD in an intuitive manner, and that reflects the different degrees of diversity of the various subsets as measured by the histograms of Euclidean distances between descriptor vectors (Fig. 3). For instance, we would expect the MC3D and MC3D-rattled to occupy roughly the same portion of space, and the MC3D-random structures to cover a much larger area, that overlaps in part with all the bulk structures; surfaces and low-dimensional structures should be at least partly separated from the bulk configurations; the organic molecules and crystals should be concentrated in a narrower region.

We extract 1,000 landmark structures from the MAD dataset using a farthest point sampling strategy^14^ using Euclidean distances between the high-dimensional feature vectors. By construction, these points serve as the most representative entries of the dataset and allow to analyze the overall coverage of the configuration space. Their UMAP^15^ and t-SNE^16^ projections (Fig. 5B,C respectively) are only partly consistent with our list of requirements. Both concentrate the MC3D-random structures and the clusters in a narrow region, and mix completely the bulk MC3D structures and the surfaces. The MC3D space is fragmented into clusters that, upon inspection, are chemically homogeneous, even though there is no reason to expect that such clustering could be exhaustive or meaningful (e.g., it would always be possible to create mixed structures that should interpolate between any pair of clusters). This tendency to “over-cluster” is a known issue with t-SNE^17^ and UMAP; in many ways, a simple PCA projection (Fig. 5A) reflects more closely our requirements, with contiguous projections of the main classes of structures, and the extremely diverse MC3D-random structures covering a large portion of the map, that overlaps only partly with the bulk inorganic materials that have less outlandish compositions. To incorporate non-linearity into the projection in a more controlled manner, we used sketch-map (SMAP)^18^, a method originally developed to analyze atomistic trajectories, that optimizes a multi-dimensional-scaling-like loss, transformed by sigmoid functions so that it aims to reproduce proximity, rather than Euclidean distance, between configurations in low and high dimension (see Section 3 for a brief overview of the method). The resulting projection (Fig. 5D) provides better separation of distinct subsets of MAD. Even though an out-of-sample projection can be performed to embed new data points on top of a sketch-map representation of landmarks, this is not very convenient – as it involves an iterative optimization for each new point. For this reason, we train a multi-layer perceptron (MLP) to reproduce the embedding of the landmarks (Fig. 5E), which matches nicely the landmark distribution from an explicit sketch-map optimization. This strategy can be viewed as a parametric extension of sketch-map, following the methodology of complementing non-linear dimensionality reduction algorithms with explicit parametric mapping^19,20^.

**Fig. 6:**
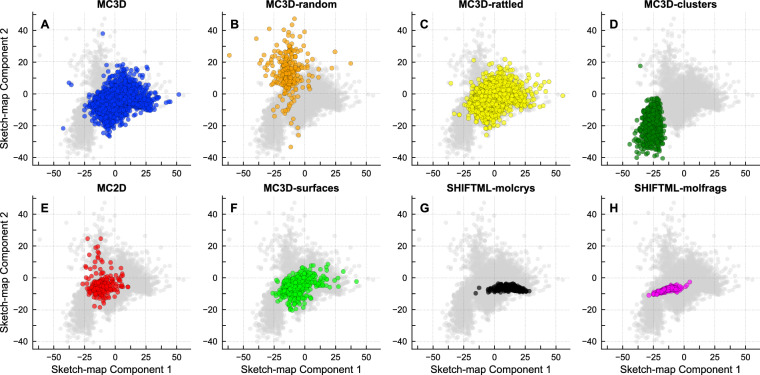
Two-dimensional sketch-map projection of subsets of MAD, built upon the last-layer features of the PET-MAD model.

We then use this MLP approximation of the sketch-map embedding to project all structures in the MAD test set onto the low-dimensional latent space. The projection of individual subsets of MAD in this latent space (Fig. 6) corresponds to that of the landmarks, and is broadly consistent with our requirements, with MC3D ideal and distorted bulk structures overlapping almost perfectly, the randomized structures covering a broad (and partly overlapping) region, molecular bulk solids covering a narrower range, and structures of lower dimensionality being progressively shifted to the bottom-left.

### Comparison with other datasets

**Fig. 7:**
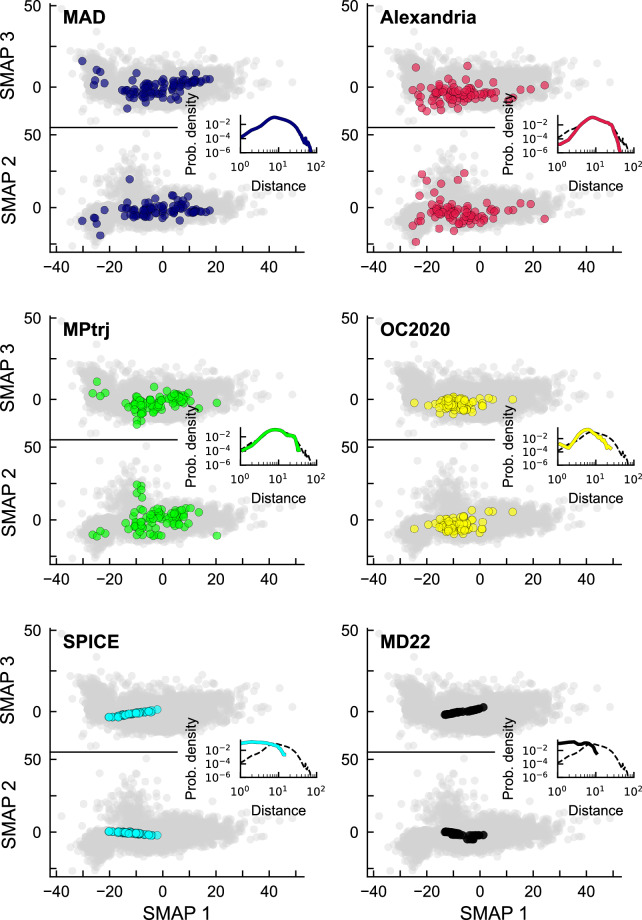
Three-dimensional projections of the MAD dataset and popular benchmarks, using MLP-trained SMAP projections of PET-MAD last-layer embeddings. The grayscale points in the background correspond to the full test subset of the MAD dataset, and the colored points in each panel to the same, small set of 85 structures randomly selected from each dataset. Insets show the histogram of the Euclidean distances between the highlighted structures in each panel, with the histogram of distances within the MAD dataset plotted for reference.

This dimensionality reduction can also be used to assess the MAD coverage relative to the existing benchmark datasets, by plotting them jointly on the same map using the same framework based on PET last-layer features and sketch-map-fitted dimensionality reduction. Figure 7 presents 2D projections of randomly selected structures from MAD and five datasets, Alexandria^8,9^, MPtrj (MACE-MP-0 Val)^2^, SPICE^21^, MD22^6^, and OC2020 (S2EF)^22^. The SPICE and MD22 datasets are narrowly focused in the center of the map, roughly in the area where the SHIFTML subsets are projected. Their low chemical diversity is also apparent in the histogram of feature-space distances, peaked at short values. OC2020 contains molecules adsorbed on surfaces, providing data to study heterogeneous catalysis, and is projected roughly in the region associated with MC3D surface subsets. In this highly-compressed projection, MPtrj and Alexandria appear to cover roughly a similar space as MAD, which however has a broader distribution as shown by the longer tail of the pairwise distance histogram in Fig. 7, which is mostly due to the MC3D-random structures (cf. the interactive visualization of projections provided in the Data Record webpage^23^). It is important to keep in mind that the embedding we used to compare the datasets is based on the PET-MAD model, which was trained on the MAD dataset. Using a different embedding – be it from a different ML model or a different training set – would likely yield different low-dimensional projections, and might emphasize structural and chemical diversity in a different way. Performing a systematic comparison of different embedding goes beyond the scope of this analysis, but the fact that the projections of different datasets are consistent with that of subsets of MAD with similar makeup substantiates the qualitative observations we make based on this specific choice of embedding.

### A generally-applicable data explorer

**Fig. 8:**
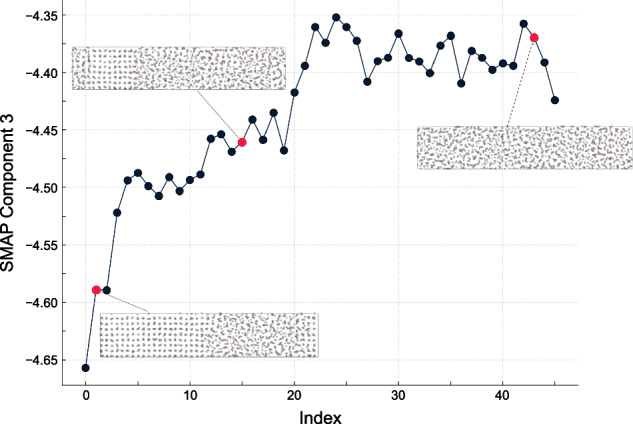
A plot of the evolution of the third sketch-map latent feature as along a MD trajectory describing the melting of Al.

The latent features optimized for the MAD representation can be computed for structures of great structural and chemical diversity, and can be used as a generally-applicable scheme to compute low-dimensional features to visualize and navigate materials datasets. We provide a simple API to do so, based on the chemiscope viewer, discussed further in Section 3. In many cases, however, one is interested in more subtle differences between structures of fixed composition, e.g., when investigating defects, chemical reactions, or phase transitions. Even though we cannot ensure that the PET-MAD latent features would be universally successful in resolving minute structural changes associated with scientifically and technologically significative transformations, we do have evidence that they *can* be rather effective. An example is shown in Fig. 8, depicting the evolution of one of the latent features as an elongated cell containing Al atoms, which is initialized in a mixed-phase state where half the atoms are in an *fcc* solid structure, and the other half are in the liquid phase. The simulation is performed slightly above the melting point of the model, and the structure slowly melts until it is entirely in the liquid state. The third latent feature (and to a lesser extent the second) is capable of resolving this transformation accurately, at least as well as carefully-crafted order parameters that have been used in the past for this kind of simulations^24^. The fact that we obtain such a resolving power as a side effect of an optimization geared towards a very different task suggests that it might be possible to develop “universal collective variables” that provide an easy-to-use foundation to kick-start the optimization of problem-specific order parameters.

## Methods

### Details of the dataset construction

In the case of MC3D, MC2D, SHIFTML-molcrys, and SHIFTML-molfrags subsets of MAD, we used previously published structures and recomputed them using a consistent set of DFT settings. For other subsets, we initialized the structure generation protocol with randomly chosen MC3D crystals and performed different transformations to increase the overall coverage of the configuration space. Rattled structures in the MC3D-rattled subset were obtained by selecting a random MC3D crystal with more than one atom in the unit cell and applying Gaussian noise to the Cartesian coordinates of each atom with a zero mean and a standard deviation equal to 20% of the corresponding covalent radii. The MC3D-random subset was built by assigning a random set of atom types to the lattice sites of a randomly chosen MC3D crystal, followed by an isotropic adjustment of the cell volume to a total atomic volume computed based on the covalent radii of the included elements. For the MC3D-surface subset, we created the surface slabs of randomly chosen MC3D crystals by cleaving them along a randomly chosen symmetrically distinct crystallographic plane with a maximum value of the Miller index (*hkl*) equal to 3 and ensuring orthogonality of the normal lattice vector to a surface plane. Finally, the structures in the MC3D-cluster subset were created by cutting a random atomic environment of 2 to 8 atoms of a randomly chosen atom from a random MC3D crystal. We discarded structures for which the DFT calculations did not converge, as well as a few outliers with forces exceeding a very large threshold (100 eV/Å for MC3D-rattled and MC3D-random and 10 eV/Å for the other subsets). Details of the first-principles calculations are provided below in Section 3. We generate a random 80:10:10 train:validation:test split of the dataset, which we recommend using to benchmark ML potentials.

To facilitate a consistent comparison with models trained against datasets (e.g., Alexandria^8,9^, MPtrj^2^ or Matbench^25,26^ that are computed using VASP and a PBE functional, using Hubbard U corrections^1^ for transition metal oxides), we also create a MAD-benchmark dataset that is computed both with MAD and MPtrj-like settings. For the MAD dataset, 50 structures were randomly sampled from each test subset and recalculated using MPtrj DFT parameters. Non-converged and outlier structures were excluded, resulting in a final set of 322 structures. Similarly, the OC2020 benchmark subset includes 89 structures, constructed by sampling 100 structures and removing non-converged cases. The Alexandria benchmark consists of 150 structures, incorporating randomly selected 50 samples from Alexandria-2D and Alexandria-3D-gopt. For the MD22 benchmark, 25 structures were randomly selected from each of the MD22’s subsets (Ac-Ala3-NHMe, AT-AT, DHA, Stachyose, AT-AT-CG-CG, Buckyball-Catcher, double-walled-nanotube). The SPICE benchmark subset consists of 100 randomly chosen neutral molecules. Finally, MPtrj (MACE-MP-0 validation subset) was reduced to 153 structures with the exclusion of 1D wire structures. For all these datasets, structures for which either type of DFT calculations did not converge were removed.

### Details of the electronic-structure reference

To maintain a consistent level of theory across the MAD dataset, all calculations were intentionally conducted without spin polarization. This choice introduces obvious errors in the description of strongly magnetic materials, avoids the likely convergence to inconsistent magnetization states, and mitigates issues related to the incorrect magnetic descriptions for elements with strong electronic correlations within spin-polarized density functional theory. The calculations were performed with Quantum Espresso v7.2^27^ compiled with the SIRIUS libraries^28^. The workflows were managed by the AiiDA framework^29–31^. We used the PBEsol functional^32^, which is designed to have better accuracy than its very similar PBE counterpart for inorganic solids, even though both are not very accurate for several classes of materials (e.g., molecular compounds). Once again, we prioritize stability and consistency for highly diverse systems over the accuracy against experiments. The behavior of semi-core electrons and their interaction with valence electrons was described using the standard solid-state pseudopotentials library (SSSP) v1.2 (efficiency set)^33^ selecting plane-wave and charge-density cutoffs (110 Ry and 1320 Ry) corresponding to the largest recommended values across the 85 elements we considered. Convergence for metallic systems was facilitated using a smearing of the electronic occupations, using the Marzari-Vanderbilt-DeVita-Payne cold smearing function^34^ with a spread of 0.01 Ry. The Brillouin zone was sampled with a *Γ*-centered grid resolution of 0.125 Å^−1^, in periodic dimensions, while non-periodic dimensions were treated with a single k-point. To prevent interaction through periodic boundary conditions in non-periodic structures, we applied the Sohier-Calandra-Mauri method^35^ for 2D systems and the Martyna-Tuckerman correction^36^ for 0D systems, with a 25 Å vacuum along non-periodic directions to ensure convergence. Additionally, a compositional baseline based on isolated atom energies was subtracted from the DFT energies to improve the numerical stability during model training. The resulting transformed energies are equivalent to the negative of the atomization energies.

These DFT settings achieved a convergence rate exceeding 95% for most of the MAD subsets, described in Section 3, with the exception of the MC3D-random structures. Due to the completely arbitrary combination of elements, these configurations had a much lower convergence rate of approximately 55%.

### Details of the dataset visualization technique

To explore the structural and chemical diversity and coverage of the MAD dataset and simplify the comparison with other atomistic datasets, we used sketch-map, a non-linear dimensionality reduction algorithm^18^ designed as an extension to multi-dimensional scaling^37^. The idea is to project high-dimensional data into a low-dimensional space while preserving *proximity* rather than the Euclidean distances between high-dimensional and low-dimensional vectors (*D*_*ij*_ and *d*_*ij*_ respectively).

More specifically, sketch-map minimizes a stress function

1 $${{\mathscr{L}}}_{sm}=\sum _{i\ne j}{w}_{ij}{(F({D}_{ij})\,-\,f({d}_{ij}))}^{2},$$

where *D*_*ij*_ and *d*_*ij*_ are the Euclidean distances between pairs of points *i* and *j* in high- and low-dimensional spaces, respectively, and *w*_*ij*_ are weights that can be included, e.g. as the product of the number of structures within the Voronoi cell of each reference landmark point. *F* and *f* are sigmoid functions which determine the classification of “far” and “near” pairs:

$$F({D}_{ij})=1\,-\,{\left[1+({2}^{A/B}\,-\,1){(\frac{{D}_{ij}}{\sigma })}^{A}\right]}^{-B/A}$$

and

$$f({d}_{ij})=1\,-\,{\left[1+({2}^{a/b}\,-\,1){(\frac{{d}_{ij}}{\sigma })}^{a}\right]}^{-b/a}.$$

The parameter *σ* controls the distance scale at which the sigmoid functions switch from 0 to 1. The parameters *A*, *B*, *a*, and *b* define the steepness and asymptotic behavior of the sigmoid transitions at short and large distances, and can be used to adjust the sensitivity of the notion of proximity in high and low dimensions.

Given the computational complexity of applying sketch-map directly to large datasets, we first selected 1,000 landmark structures from the MAD test set using farthest-point sampling, a method that iteratively chooses structures to maximize coverage of configuration space^14^. The sketch-map projection was then performed for the landmarks with a sigmoid transformation defined by parameters: *σ* = 7, *A* = 4, and *B* = 2 for the high-dimensional space; and *a* = 2, *b* = 2 for the low-dimensional space, following the hyperparameter selection methodology described in^38^. The landmark projections are initialized to a 2D PCA, followed by a sequence of local and global optimization steps. Finally, an iterative optimization is performed including a third low-dimensional component to allow for a more descriptive 3D representation. After having obtained a 3D projection of the landmarks, we train a simple neural network to reproduce the sketch-map embedding in a simpler and less computationally demanding way. We use a Multi-Layer Perceptron (MLP) architecture, with three hidden layers with ReLU activation functions, to map high-dimensional PET-MAD descriptors to 3D sketch-map coordinates. We trained the model on the landmarks, using an 80:20 train-validation split and using SmoothL1Loss^39^ to assess the error in the projection. The MLP was then applied to project the remaining points from the MAD validation split and the benchmark datasets.

**Fig. 9:**
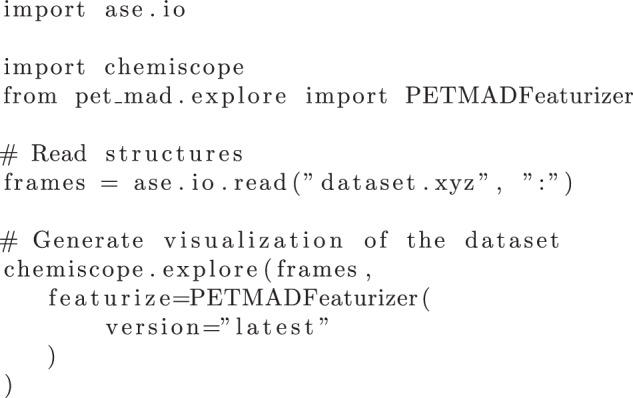
Example of Python code for visualizing a dataset using Chemiscope with PET-MAD descriptors mapped to sketch-map coordinates. Here, frames represent a list of ASE^48^ compatible input structures, and featurizer extracts the PET-MAD descriptors, which are then mapped to the sketch-map coordinates using the trained MLP.

To visualize the resulting projections and analyze their compositions, we used Chemiscope^40,41^, a visualization tool that allows users to interactively explore atomistic structures and their properties. It enables one to inspect the low-dimensional projections and associated structures and their properties. Figure 9 shows a code snippet demonstrating how to use Chemiscope to visualize a new dataset with the PET-MAD model.

## Data Record

The dataset is made available as a record^23^ within the Materials Cloud^3^ Archive, which is a FAIR repository dedicated to materials-science simulations. The data is stored in the format of AiiDA archive files, which contain the full provenance graph of the calculations and can be accessed using the tools provided by the AiiDA package^29^. In addition to this monolithic database, we extract parts of the data in a more compact and easier-to-access format (the extended XYZ format, that stores energies, stresses, and lattice parameters in the header, and atom types, positions, and forces as space-separated entries). Cartesian coordinates, energies, forces, and stresses are given in Å, eV, eV/Å, and eV/Å^3^, respectively.

More specifically, we provide:

- The MAD dataset, with an 80:10:10 train:validation:test split as used in training the PET-MAD model: *mad-train.xyz*, *mad-val.xyz*, *mad-test.xyz*. The subsets are indicated as a field in the header.
- AiiDA database archives for each subset of the MAD dataset: *mad-mc3d.aiida*, *mad-mc3d-rattled.aiida*, *mad-mc3d-random.aiida*, *mad-mc3d-clusters.aiida*, *mad-mc3d-surfaces.aiida*, *mad-mc2d.aiida*, *mad-shiftml-molcrys.aiida*, *mad-shiftml-molfrags.aiida*.
- The MAD benchmark dataset, containing a selection of MAD test, MPtrj, Alexandria, SPICE, MD22, and OC2020 datasets, computed with both MAD DFT settings and MPtrj DFT settings. These are provided as two separate files: *mad-bench-mad-settings.xyz*, *mad-bench-mptrj-settings.xyz*. The parent dataset is indicated as a field in the header.
- A zipped folder *mad-aiida-aux.zip* with AiiDA database archives for the MAD benchmark calculations, as well as some auxiliary calculations done for the MAD dataset.
- A CSV table *atomic-energies.csv* with the energies of isolated atoms used as the energy baseline in MAD calculations.

To facilitate visualization, we also provide chemiscope visualization files corresponding to Figs. 5, 6, and 7– containing 2D or 3D latent space projections, as well as energies for each structure. These files, named mad-landmarks.chemiscope.json.gz, mad-subsets.chemiscope.json.gz, and mad-bench.chemiscope.json.gz, respectively, can be viewed and interacted using the Chemiscope web interface at http://chemiscope.org (or directly via custom links from the Materials Cloud Archive record page) or programmatically via the Chemiscope API using chemiscope.show_input("mad- subsets.chemiscope.json.gz") in a Jupyter notebook.

More details on how to interact with the data are provided in the data record page^23^.

## Technical Validation

The quality of the dataset in terms of its internal consistency and configuration space coverage was tested by training the PET-MAD model and comparing the computational experiment results against those of bespoke PET models trained on problem-specific datasets^13^. First, the tight convergence and consistency of the DFT calculations in the MAD dataset allowed us to obtain low training error values for the PET-MAD model, around 5 meV/atom for energy prediction. This value serves as an initial indication of the quality of the training and an upper limit on the intrinsic unlearnable noise present in the training data. In all showcase studies, including calculations of ionic conductivity in Li_3_PS_4_, the melting point of GaAs, the dielectric response in BaTiO_3_, surface segregation in the CoCrFeMnNi high-entropy alloy, quantum nuclear effects in water, and NMR crystallography, the performance of the universal PET-MAD model, trained on the MAD dataset, was sufficiently close to (and sometimes indistinguishable from) the results of bespoke PET models. This observation not only indicates the PET model’s good generalizability, but also demonstrates how the high diversity of the dataset makes it possible to obtain stable, reliable simulations across a variety of material classes and advanced modeling techniques.

## Data Availability

All data generated in this study have been deposited in the Materials Cloud Archive database under accession code materialscloud:2025.145. All the computational workflows presented in this study can be analyzed and reproduced using the AiiDA database files distributed via the aforementioned data record.

## Code availability

All the data in this work was generated using the open-source AiiDA workflow manager, accompanied by aiida-quantumespresso extension for running the calculation using the Quantum Espresso code, as well as the aiida-submission-controller extension for managing the calculations in the HPC clusters queue. All the code for the core part of AiiDA is available on GitHub: https://github.com/aiidateam/aiida-core. Both mentioned extensions can also be found on GitHub: https://github.com/aiidateam/aiida-quantumespresso and https://github.com/aiidateam/aiida-submission-controller. The PET-MAD model, together with the PET-MAD featurizer required for datasets visualization, is available in the GitHub repository: https://github.com/lab-cosmo/pet-mad.
